# The Expression Pattern of Surface Markers in Canine Adipose-Derived Mesenchymal Stem Cells

**DOI:** 10.3390/ijms22147476

**Published:** 2021-07-12

**Authors:** Nina Krešić, Marina Prišlin, Dunja Vlahović, Petar Kostešić, Ivana Ljolje, Dragan Brnić, Nenad Turk, Andrija Musulin, Boris Habrun

**Affiliations:** 1 Virology Department, Croatian Veterinary Institute, Savska Cesta 143, 10 000 Zagreb, Croatia; prislin@veinst.hr (M.P.); brnic@veinst.hr (D.B.); 2 Department of Veterinary Pathology, Faculty of Veterinary Medicine, University of Zagreb, Heinzelova 55, 10 000 Zagreb, Croatia; dvlahovic@vef.hr; 3 Surgery, Orthopaedics and Ophthalmology Clinic, Faculty of Veterinary Medicine, University of Zagreb, Heinzelova 55, 10 000 Zagreb, Croatia; kostesic@vef.hr (P.K.); musulin@vef.hr (A.M.); 4 Veterinary Clinic for Small Animals Buba, Dore Pfanove 11, 10 000 Zagreb, Croatia; ivana.ljolje@gmail.com; 5 Department of Microbiology and Infectious Diseases with Clinic, Faculty of Veterinary Medicine, University of Zagreb, Heinzelova 55, 10 000 Zagreb, Croatia; turk@vef.hr; 6 Department for Bacteriology and Parasitology, Croatian Veterinary Institute, Savska Cesta 143, 10 000 Zagreb, Croatia; habrun@veinst.hr

**Keywords:** stem cells, in vitro cultivation, immunophenotype, gene expression, flow cytometry, canine

## Abstract

The influence of cultivation on the expression pattern of canine adipose-derived mesenchymal stem cells (cAD-MSCs) surface markers, contributing to, among others, the promotion of growth, proliferation, differentiation and immunomodulatory mechanisms of an excellent therapeutic, is still unknown. To fill the gap, we investigated CD90, CD44, CD73, CD29, CD271, CD105, CD45 and CD14 patterns of expression at the protein level with flow cytometry and mRNA level using a real-time polymerase chain reaction array. Gentle variations of expression occurred during cultivation, along with increased CD90, CD44 and CD29 expression, low and decreasing CD271 and CD73 expression and a decrease of initially high CD105. As expected, CD45 and CD14 were not expressed by cAD-MSCs. Interestingly, we discovered a significant decrease of CD73 expression, compared to early (P1–P3) to late (P4–P6) passages, although the CD73 gene expression was found to be stable. The percentage of positive cells was found to be higher for all positive markers up to P4. As CD73′s one important feature is a modulation from a pro-inflammatory environment to an anti-inflammatory milieu, the expression of CD73 in our conditions indicate the need to consider the time cells spend in vitro before being transplanted into patients, since it could impact their favourable therapeutical properties.

## 1. Introduction

The International Society for Cellular Therapy (ISCT) established minimum criteria for the definition of MSCs: adherence to tissue culture plastic; multipotency as demonstrated by in vitro differentiation into osteoblasts, adipocytes and chondroblasts; expression of surface markers CD73, CD90 and CD105 and negative for CD34, CD45, CD14 or CD11b, C79α or CD19 and HLA-DR [1]. Many studies demonstrated that cells meeting the ISCT criteria possessed heterogeneous phenotypes and functionalities, heavily influenced by culture conditions [2].

The goal of successful expansion in vitro is to maintain stem cell phenotype and show repeated self-renewal, i.e., undergo extensive proliferation while maintaining their multipotent differentiation potentials [3]. The widespread lack of culture condition homogeneity between laboratories has made it difficult to reach unequivocal consensus even on basic MSCs properties, leading to discrepancies at the basic research level and adding biases to the evaluation of stem cell-based clinical study outcomes [2]. Despite that, isolated culture-expanded cells have been applied in both preclinical and clinical settings in animals with variable but mostly positive results. Further intensive research on the cell properties and application are certainly needed.

Cultivation in vitro also affects the characteristic MSCs membrane markers used to confirm the identity of isolated cells. Therefore, the expression of established MSCs markers, clusters of differentiation (CD), may be influenced by factors secreted by accessory cells in the initial passages (P). Additionally, the in vitro expression of some MSCs markers does not always correlate with their expression patterns in vivo [4].

The importance of MSCs markers is the promotion of growth, proliferation, differentiation and survival of cells in stem cell habitat. Currently, there is no unique cell marker capable of solely isolating and defining MSCs, but THY1 (CD90), a glycoprotein present in the MSCs membranes, is related to the state of cellular non-differentiation [5]. CD90 is an anchored cell surface protein usually expressed on thymocytes, MSC, natural killer cells, neurons, endothelial cells, renal glomerular mesangial cells, follicular dendritic cells, fibroblasts and myofibroblasts. It has been found to regulate cell adhesion, migration, apoptosis, axon growth, cell–cell and cell-matrix interactions, T-cell activation and fibrosis [6]. Besides the specific expression pattern and functions of CD90 that were described in normal tissues, increasing evidence is currently highlighting the possible involvement of CD90 in cancer [7].

Another marker involved in migration and adhesion is CD44, a glycoprotein widely expressed on the surface endothelial cells, epithelial cells, fibroblasts, keratinocytes and leukocytes. CD44 has important functions in cell–cell and cell-matrix interactions, including proliferation, hematopoiesis and lymphocyte activation, homing and extravasation [8]. CD44 is essential for maintaining cartilage homeostasis, influences the production of collagen II and aggrecan and influences the chondrodifferentiation of amniotic MSCs [9]. CD44 has been implicated in cancer, arthritis, interstitial lung disease, vascular disease, wound-healing and infections by pathogens [8]. 

CD73, ecto-5-nucleotidase, is a membrane protein that dephosphorylates extracellular AMP to bioactive adenosine that leads a shift from an ATP-driven pro-inflammatory environment to an anti-inflammatory milieu [10]. CD73 expression is heterogeneous in MSCs derived from various sources: with MSCs from human umbilical cord blood at the highest and bone-marrow-derived MSC at the lowest, suggesting that nonuniform expression of CD73 is a ubiquitous phenomenon in the MSC pool [4]. One important feature of CD73-positive cells is their ability to modulate the immune response. Transplantation of CD73-positive cells suppressed fibrosis and inflammation in pulmonary fibrosis model mice. Although the clinical translation of these results requires further detailed research, CD73-positive cells might provide a new strategy for treating and managing pulmonary fibrosis. Furthermore, interstitial pneumonia after COVID-19 infection should be considered for the candidate disease [11].

CD29, or integrin β1, is a cell surface receptor involved in the interaction of cells with extracellular matrix proteins such as collagen, laminin and fibronectin. Integrin family members are membrane receptors involved in cell adhesion and recognition in a variety of processes, including embryogenesis, hemostasis, tissue repair, immune response and metastatic diffusion of tumour cells [12]. Among all, it has been shown that CD29 is strongly expressed by adipocyte progenitors [13,14]

CD271, low-affinity nerve growth factor receptor (LNGFR), nerve growth factor receptor (NGFR) or p75NTR (neurotrophin receptor), belongs to the tumour necrosis factor superfamily. CD271 has been proposed as a versatile marker to selectively isolate and expand multipotent mesenchymal stem cells with immunosuppressive and lymphohematopoietic-engraftment-promoting properties. In the case of bone marrow or adipose tissue, CD271 could be considered a suitable marker for MSCs isolation [15].

Endoglin (ENG), or CD105, is a type I membrane glycoprotein located on cell surfaces and is part of the TGF beta receptor complex [16]. Endoglin may be involved in the cytoskeletal sorganisation affecting cell morphology and migration [17]. Bearden et al. (2017) found that ~60% of cAD-MSCs were positive for CD105 [18]. Furthermore, the expression of CD105 in murine and human AD-MSCs was induced by the exposure of cells to culture plastic and was further affected by passage number and confluence [19].

The expression patterns of surface markers specific for canine adipose-derived MSCs (cAD-MSCs) and changes in the percentages of positive cells during cultivation and serial passaging are still unknown. Thus, we aimed to analyse the expression pattern and percentage of positive cells for selected CDs (CD90, CD44, CD73, CD29, CD271, CD45 and CD14) during in vitro cultivation. Furthermore, we investigated changes in the expression of selected markers on mRNA level between P3 and P6 using array technology. We performed the comparison of surface marker expression pattern and positivity percentage between early (P1–P3) and late passages (P4–P6). The study encompassed freshly isolated cAD-MSCs originating from young, healthy donors. For the first time, comprehensive analysis of cAD-MSCs markers dynamics during in vitro cultivation is presented with a final contribution to understanding their therapeutical potential.

## 2. Results

### 2.1. cAD-MSCs Terminate Proliferation at the Sixth and Successfully Differentiate in the Third Passage

Since cAD-MSCs characteristics during in vitro expansion are heavily influenced by culturing conditions, we aimed to reveal the expansion, functional and immunophenotype characteristics of cells in each passage until their proliferation arrest.

For the cAD-MSCs isolation, we employed our previously published protocol [20] based on collagenase digestion of adipose tissue samples which resulted in the successful isolation of cAD-MSCs from all donors. Similarly to our previous results, isolated cells were spindle-shaped, and successfully proliferated in vitro up to P6. Although the cells of most donors managed to survive only up to P6, the 13/20 cells showed excellent proliferative capacity and reached P8. During cultivation time, well-known changes in cellular morphology and proliferation capacity occurred [21,22], resulting in a prolonged time needed to reach 80% confluence.

**Figure 1:**
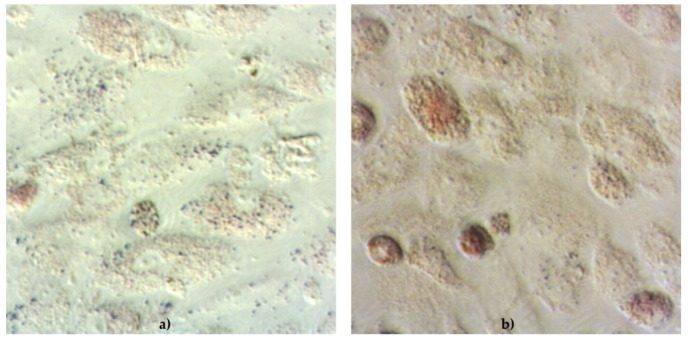
Functionality of canine adipose-derived mesenchymal stem cells (cAD-MSCs) demonstrated by successful differentiation into adipocytes. Microscopic images (40×) of cAD-MSCs after adipodifferentiation stained with Oil O Red for detection of lipid droplets within cell. Cells cultivated for 21 days in basal medium (negative control) (**a**) and stained with Oil O Red, showing lack of red staining. cAD-MSCs cultivated for 21 days in StemMACS AdipoDiff Media (**b**) successfully differentiated into adipocytes. When stained with Oil O Red, accumulated lipid droplets show high-intensity red staining within cell.

**Figure 2:**
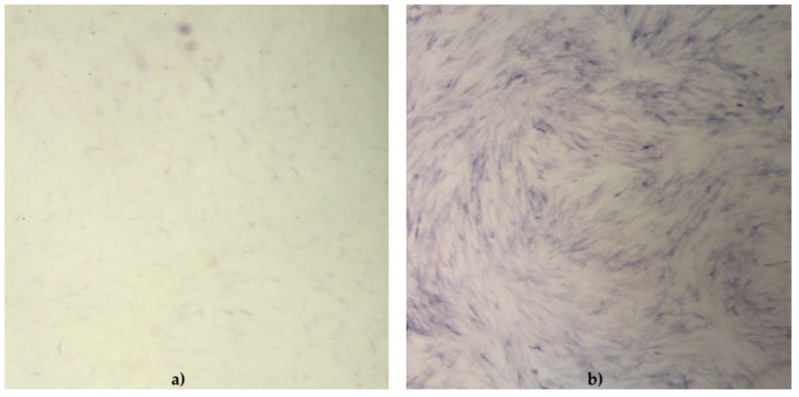
Functionality of canine adipose-derived mesenchymal stem cells (cAD-MSCs) demonstrated by successful differentiation into osteoblasts. Microscopic images (18×) of cAD-MSCs after osteodifferentiation. Cells were stained with SIGMAFAST(TM) BCIP (R)/NTB substrate to detect alkaline phosphatase activity. Cells were cultivated for 21 days in basal medium (negative control) (**a**) and stained with SIGMAFAST(TM) BCIP (R)/NTB, showing low-intensity staining. cAD-MSCs cultivated for 21 days in StemMACS OsteoDiff Media (**b**) and stained with SIGMAFAST(TM) BCIP (R)/NTB, showing high-intensity staining for alkaline phosphatase activity. The cells were examined under a stereomicroscope (Stereo Discovery, V20, CL1500 ECO, Zeiss).

**Figure 3:**
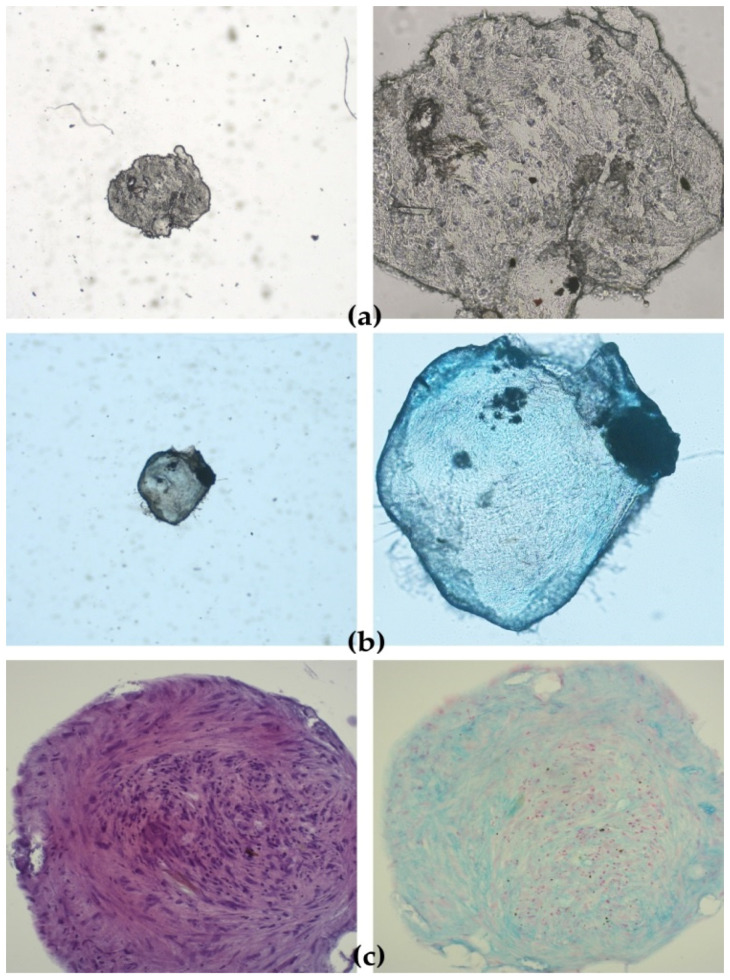
Functionality of canine adipose-derived mesenchymal stem cells (cAD-MSCs) demonstrated by successful chondrodifferentiation. Microscopic images of spheroids cultivated for 24 days in basal medium (negative control) stained with Alcian Blue showing lack of staining for extracellular matrix (ECM) aggrecan, 5× and 20× (**a**). Spheroids cultivated for 24 days in StemMACS ChondroDiff Media, stained with Alcian Blue, showing staining for ECM aggrecan, 5× and 20× (**b**). Images of histological sections of paraffin-embedded spheroids stained with hematoxylin-eosin (H&E) and Alcian Blue 20× (**c**).

The functionality of cAD-MSCs in vitro was well preserved, as demonstrated by successful differentiation into adipocytes (Figure 1), osteoblasts (Figure 2) and chondrocytes (Figure 3). Representative differentiation results from one experiment are shown in Figure 1, Figure 2 and Figure 3.

During the differentiation experiments, microscopic changes in cellular phenotype became visible in the form of lipid droplets in the cytoplasm of rounded cells induced to differentiate into adipocytes.

Within this study, we improved the ability to detect lipid droplets accumulated within the cell during adipose-differentiation and reduced loss of lipid commonly occurring with the use of methanol. Although alcohol fixatives are considered for better-preserved morphology [23], the use of formaldehyde overcome the loss of lipid from the cells. Unfortunately, both of them are toxic products, methanol neurotoxic and formaldehyde mutagen.

The cell phenotype in osteodifferentiating wells was in the form of elongated cells. Cells in negative control wells preserved characteristic undifferentiated morphology.

### 2.2. CD73 Expression Significantly Changed between Early and Late Passages

Driven by the lack of information on the changes in expression of specific markers, we analysed their pattern of expression during cultivation in standard conditions. Given the very important function of each of these markers, it is to be expected that changes in their expression could affect the therapeutic properties of cells that have been propagated in vitro.

Besides surface markers expression pattern, we aimed to analyse the changes occurring in the cell population during extensive passaging in vitro reflected as changes of the percentage of positive cells. To achieve our aims, we performed serial investigations on protein and mRNA levels. This study, for the first time, brings comprehensive immunophenotyping of cAD-MSCs comprising a set of eight characteristic markers (CD90, CD44, CD73, CD29, CD271, CD105, CD45 and CD14). To avoid any influence on the pattern of expression and changes within the cellular population, we analysed cells without freezing. During the analysis, we managed to maintain high cellular viability as the manipulation with cells was very careful, and reading was performed immediately after the staining and washing step.

**Figure 4:**
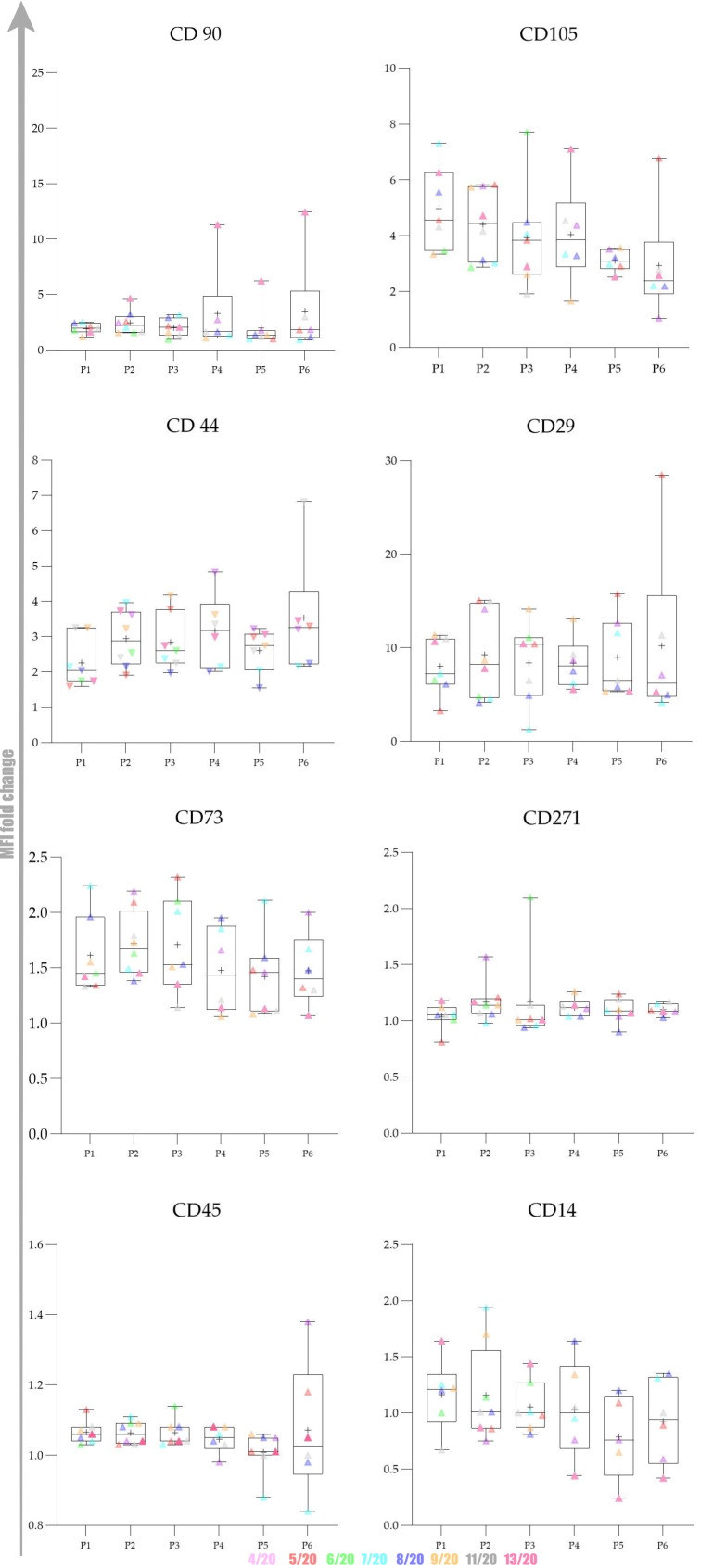
Canine adipose-derived mesenchymal stem cells (cAD-MSCs) surface markers expression pattern during in vitro cultivation. cAD-MSCs. The expression pattern was analysed by calculation of fold change of median fluorescence intensity (MFI). The results are based on eight biological replicates, young female canines referred to as elective surgery procedures with no obvious signs of disease. Flow cytometry assessment of positive and negative cAD-MSCs markers showed no statistically significant changes in the expression of the following positive markers: CD90, CD44, CD29, CD105 and CD271. A statistically significant change in expression was observed for CD73 between early (P1–P3) and late (P4–P6) passages. The *X*-axis represents the passage number (from P1 to P6), whereas the *Y*-axis displays the average fold change of MFI. MFI fold change was calculated by dividing the median of the appropriate antibody with the median of isotype control. The Whisker box plot was created to present the dynamics of changes between passages. Centerlines denote the median, plus the mean, box limits indicate the twenty-fifth and seventy-fifth percentiles; whiskers represent the maximum and minimum of the acquired values. Individual values are shown in colours to reveal changes for each donor included in the study. Donor 4/20 is presented in light pink, 5/20 in red, 6/20 in green, 7/20 in light blue, 8/20 in purple, 9/20 in orange, 11/20 in grey and 13/20 in dark pink.

**Figure 5:**
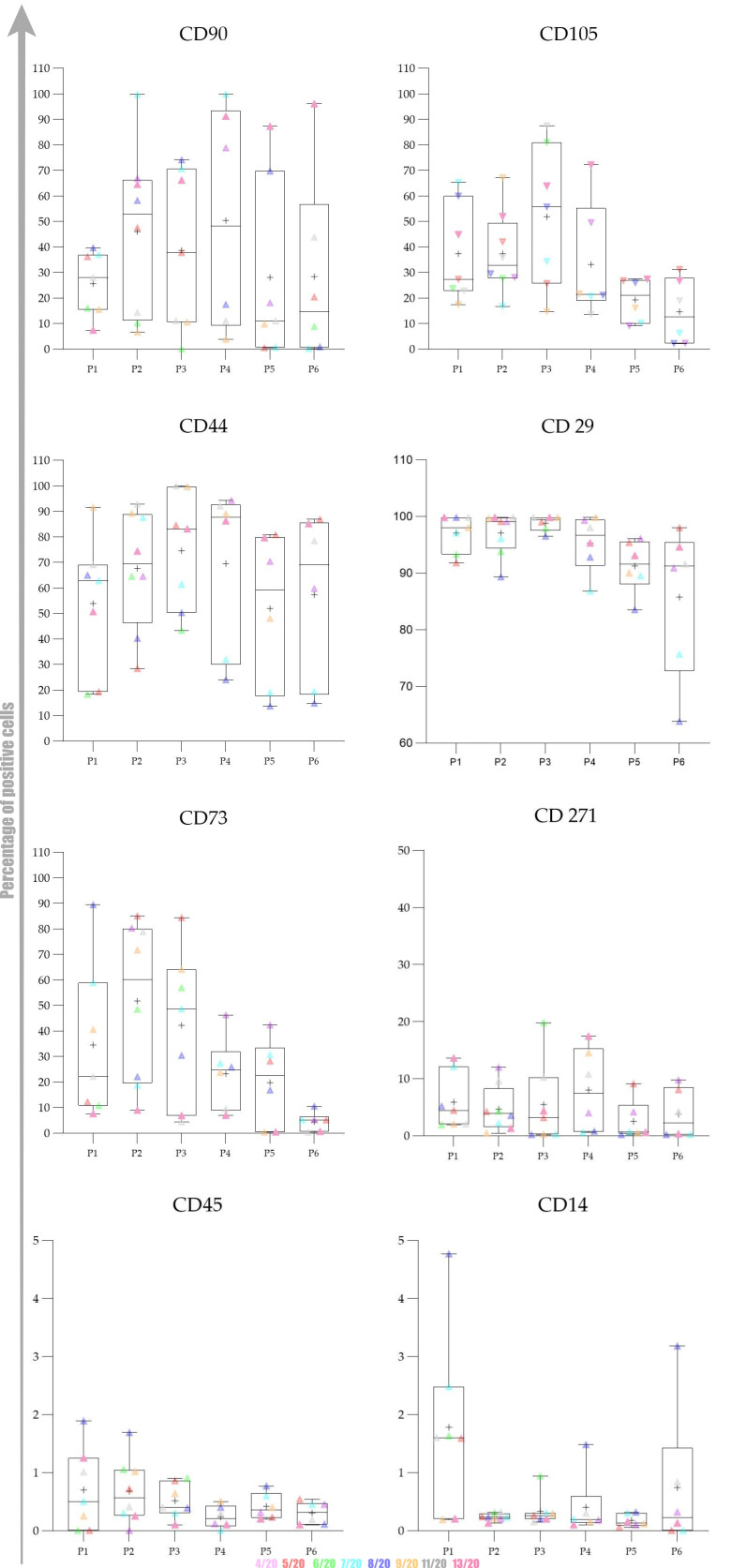
Changes in the percentage of positivity of canine adipose-derived mesenchymal stem cells (cAD-MSCs) during in vitro cultivation. The results are based on eight biological replicates, young female canines referred to an elective surgery procedure with no obvious signs of disease. Flow cytometry assessment of surface marker positive cAD-MSCs showed no statistically significant changes in the percentage of positive cells for the following markers: CD90, CD44, CD29, CD105 and CD271. Statistically significant changes were observed for CD73 between early (P1–P3) and late (P4–P6) passages. The *X*-axis represents the passage number (from P1 to P6), whereas the *Y*-axis displays the average positivity percentage. The Whisker box plot was created to present the dynamics of changes between passages. Centerlines denote the median, plus the mean, box limits indicate the twenty-fifth and seventy-fifth percentiles; whiskers represent the maximum and minimum of the average positivity percentage. Individual values are shown in colours to reveal changes for each donor included in the study. Donor 4/20 is presented in light pink, 5/20 in red, 6/20 in green, 7/20 in light blue, 8/20 in purple, 9/20 in orange, 11/20 in grey and 13/20 in dark pink.

**Figure 6:**
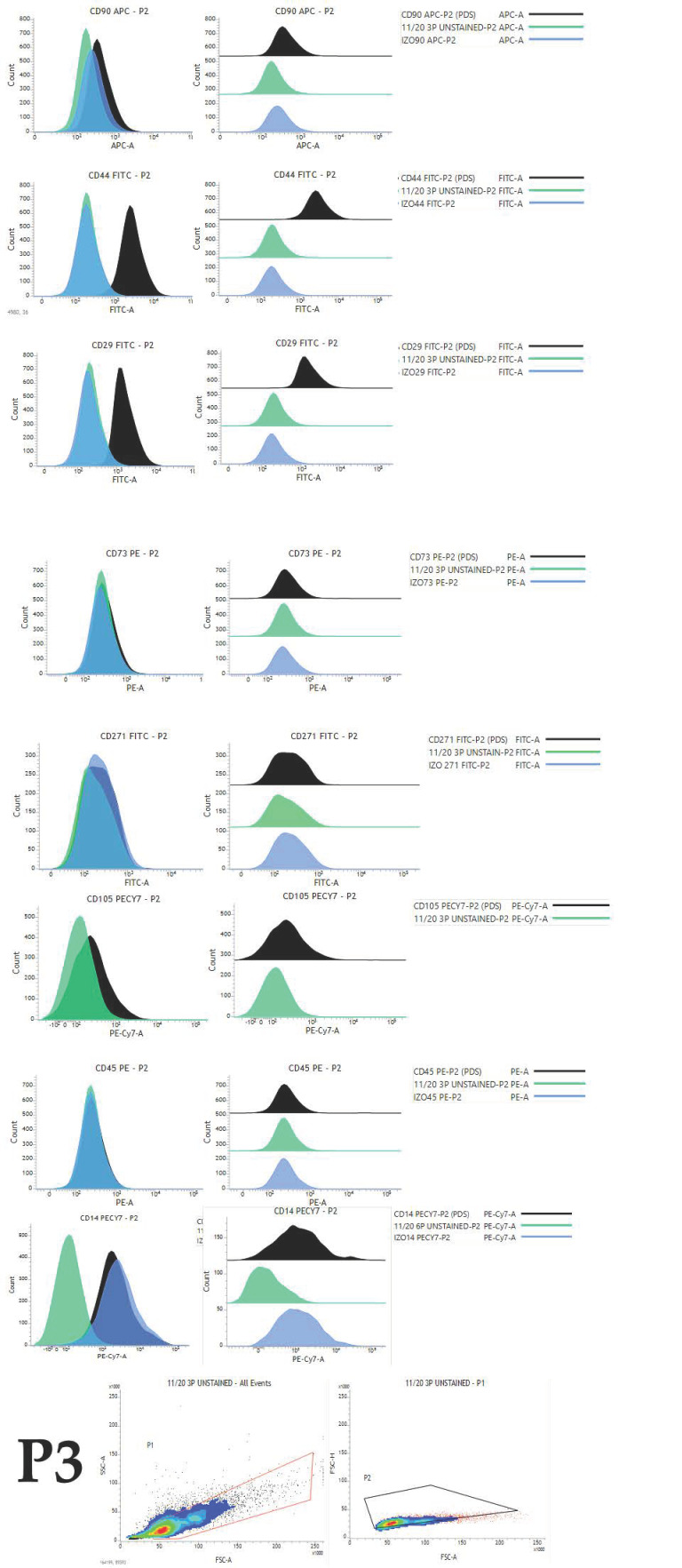
Histogram representation of immunophenotyping results obtained by flow cytometry analysis of cells in passage 3 originating from representative young female canine donor. Histogram presents overlayed unstained population (green), appropriate isotype control (blue) and fully stained population of cells (black).

**Figure 7:**
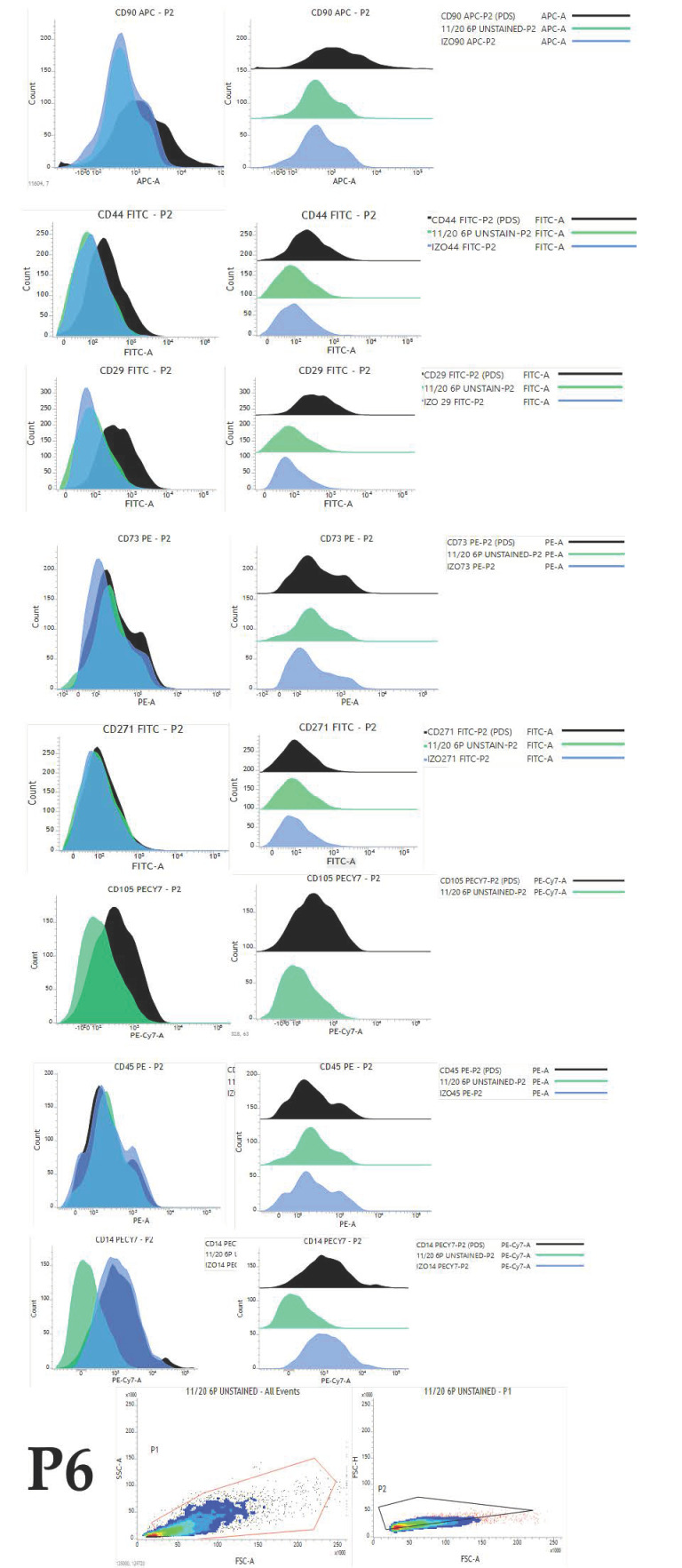
Histogram representation of immunophenotyping results obtained by flow cytometry analysis of cells in passage 6 originating from representative young female canine donor. Histogram presents overlayed unstained population (green), appropriate isotype control (blue) and fully stained population of cells (black).

Overall expression results are presented in Figure 4. Changes in the percentage of positive cells between passages are shown in Figure 5, Figure 6 and Figure 7.

The obtained results encouraged us to investigate whether there was any statistically significant difference between early (P1–P3) and late passages (P4–P6). We observed that cells passaged up to P4 had a more favourable phenotype as investigated by microscopy and the expression of specific markers. Furthermore, we also observed that the percentage of positive cells was higher in early passages. No statistically significant differences in expression and percentage of positive cells were found between early (P1–P3) and late passages (P4–P6) for all of the analysed markers except for CD73. Changes in expression of CD73 were found to be statistically significant (*p* = 0.0049) as well as changes in percentage of CD73^+^ cells (*p* = 0.0023) Change in percentage CD105^+^ was found to be almost statistically significant (*p* = 0.054).

### 2.3. Gene Expression

We also aimed to investigate gene expression changes between early (P3) and late passage (P6). We used commercially available array as it enables quick, reliable gene expression analysis. The array encompasses laboratory-verified qPCR assays, with integrated, patented controls to ensure a successful experiment. The results obtained by analysis of RNA integrity and purity matched the criteria needed for the downstream application of RNA prescribed by the array manufacturer (A_260_:A_230_ ratio greater than 1.7; A_260_:A_230_ ratio 1.8–2.0; concentration determined by A_260_ > 40 μg/mL). Integrity analysis showed the absence of ribosomal RNA degradation with a 28 S/18 S rRNA amount ratio of approximately 2 for all samples.

Our gene expression analysis revealed upregulation of CD44, CD29 and CD45, downregulation of CD90, while CD73 expression was stable: Figure 5 and Figure 6. Ct values, *p* values and fold regulation are demonstrated in Table 1.

## 3. Discussion

Since stem cell therapy involves cellular manipulation and in vitro cultivation, it is necessary to reveal relevant surface markers expression dynamics during cultivation to predict how cultivation affects the expression and consequently stem cell desirable therapeutic properties.

The influence of cultivation on morphology, phenotype and gene expression changes is the subject of investigation in human MSCs [2,24,25]. However, similar studies involving canine stem cells are lacking. This study, for the first time, revealed changes in the percentage of positivity and surface markers expression dynamics of cAD-MSCs during cultivation. By that, we contributed to their proper cAD-MSCs characterisation and demonstrated the timeframe in which cAD-MSCs cultivated in vitro possess optimal phenotypes for transplantation.

The role of canines as companion animals is on the rise, and research on cAD-MSCs is important as it will provide basic knowledge about cAD-MSCs properties. It will make the foundation for future studies, basic and applied, to ensure excellent stem cell therapies for canines for various conditions.

The expression of CD90/Thy-1 is demonstrated in different cells at different locations in the body but also highly expressed in the aggressive high malignancy grade basal-like breast tumour cell line (Hs578T), pointing to this molecule as a promising breast tumour marker [26]. CD90 is also highly expressed in all MSCs, which is related to the undifferentiated status of MSCs, and a decrease in CD90 level can be correlated with the temporal lineage commitment in vitro [27,28]. The expression pattern for CD90 in canine umbilical cord MSCs was negatively correlated with the number of passages [29]. CD90 expression in our study gently varied during cultivation but generally slightly increased at the protein level. The increase in P4 and P6 was mainly due to high fold change values in P4 and P6 in cells originating from donor 13/20, the oldest donor among all in the study whose cells managed to reach P8. The average fold change of CD90 in P6 of remaining donors in the study was very low (1.74). The reasons for such variable CD90 expression between 13/20 and the rest of the donors can be explained with intraspecies variability, an important feature of the canine population. The origin of adipose tissue probably could not have an impact on CD90 expression since we found no correlation between MFI values and the area of cAD-MSCs origin (ligament falciform, mesenteric and periovarian area).

Since CD90 appears to influence cell proliferation, differentiation, migration and survival [28], the decrease in its expression during cultivation in cells originating from the other seven donors could be contributing to cellular proliferation arrest that occurred in P6 as well as prolonged proliferation in the case of 13/20 donor.

Downregulation of CD90 gene expression is in line with flow cytometry data after exclusion of 13/20 extreme donor values. Such a discrepancy between CD90 expression at protein and mRNA level as in the case of 13/20 is not a surprise due to the known poor overall correlation between mRNAs and their protein products. The demonstrated decrease in the percentage of CD90^+^ cells is not a surprise since it has already been reported in our previous research [20].

High CD44 expression in our conditions is in line with the high and rising expression of CD44 in canine umbilical cord MSCs [29] and human AD-MSCs (hAD-MSCs) [25]. Gharibi et al. (2014) reported persistent expression of surface markers including CD146, CD105, CD44, CD90 and CD71 by flow cytometry throughout, and expression of these putative stem cell markers persisted even after the loss of differentiation potentials. The expression of CD44 is important from the aspect of possible influence on chondrodifferentiation. Newly published evidence shows that the deficiency of CD44 could inhibit the formation of type II collagen and significantly decrease the production of aggrecan, and CD44 could affect the differentiation of hAD-MSCs into chondrocytes [9]. High expression of CD44 in later passages could indicate preservation of important stem cell features that this molecule is implied to be involved with, such as homing and proliferation. Our gene expression results revealed CD44 upregulation, which can be considered a beneficial finding.

CD45 is a hematopoietic lineage-restricted antigen that is expressed on all hematopoietic cells except for some mature cell types [30]. With passages, the culture of mesenchymal stem cells is decontaminated, and CD45 expression decrease. The expression of CD45 at the mRNA level was very low, which is in line with our protein expression results.

CD73 expression in different subpopulations has the highest inconsistency, indicating that CD73 is a sensitive characteristic of MSCs [31]. Biological characteristics and differentiation potential of CD73^+^ and CD73^−^ cAD-MSCs show that CD73^+^ AD-MSCs are mainly small-sized cells, whereas CD73^−^ AD-MSCs are big-sized cells; both subpopulations can equally differentiate into adipocytes and osteoblasts in vitro [31].

According to our results, cAD-MSCs show low CD73 expression that significantly decreased during in vitro cultivation. The expression pattern of CD73 in our conditions indicate the need to carefully consider the time cells spent in vitro and at which cells are transplanted into patients or model animals, since it could impact their favourable properties and therapeutical effects.

We demonstrated that cAD-MSCs express CD271 at low levels similarly to placenta-derived MSCs while it is not expressed in the synovial membrane [32]. Barilani et al., 2018, detected the highest number of CD271^+^ MSCs soon after isolation in serum-based culture conditions. On the contrary to cAD-MSCs, human bone marrow, adipose-derived and periodontal ligament MSCs show a high level of CD271 expression. CD271^+^ AD-MSCs were proposed as the primary choice for tissue regeneration and autologous stem cell therapies in older subjects [33].

Our data also revealed high and rising expression of CD29 during cultivation time. CD29 proved to be highly expressed on cAD-MSCs, and more than 95% of cells in culture expressed CD29, similar to bone marrow and hAD-MSCs [34].

CD105 expression by cAD-MSCs demonstrated here is in line with murine and hAD-MSCs, where it was induced by exposure of cells to tissue culture plastic and was further affected by passage number and confluence [19].

This study revealed no statistically significant changes in the expression pattern of selected markers or percentage of positive cAD-MSCs, except for CD73. Generally, early passages seem to have a more favourable immunophenotype. However, there are reports on preserved immunophenotype during cultivation up to P6 for hAD-MSCs [25]. More research would be beneficial to reveal the real impact of cultivation on the nature of cultivated cells.

Furthermore, there is a trend towards early termination of AD-MSCs cultures in vitro, usually before the second or third passage. This short-term culturing may, in turn, lead to low, suboptimal cell titer for downstream applications of AD-MSCs. Those obstacles need to be overcome for using cAD-MSCs in the best condition for therapy, and by that, raising the positive therapeutic effect in canines.

In conclusion, one of the most important insights this study brought is the timeframe in which cAD-MSCs cultivated in vitro possess optimal immunophenotype for use in therapy.

The important limitation of the study is the lack of in vivo implication that is clearly needed. The investigations of stem cell effectiveness are obligatory as it reliably provides evidence on the cellular effectiveness and reveals whether demonstrated changes in expression affect their therapeutical properties.

Nevertheless, the peculiarities of cells originating from older donors also remain to be addressed in future research.

## 4. Materials and Methods

### 4.1. Ethics

The animal protocols used in this work were evaluated and approved by the by Veterinary Ethics Committee at the Faculty of Veterinary Medicine, University of Zagreb, approval code 640-01/20-17/10, 20 February 2020 and 640-01/20-17/55, 28 September 2020 and Ethics Board of Croatian Veterinary Institute, approval code Z-IV-4-2022/19, 9 May 2019. Owners of all canine donors included in this study provided written informed consent for the use of the biological materials of their pets in research.

### 4.2. Animals

This study encompasses 8 healthy canine (*Canis lupus familiaris*) female donors aged 6–38 months referred to elective surgical procedures. The donors’ details are presented in Table 2.

Considering the diversity of the canine population, we aimed to eliminate other possible influencing factors such as age and sex and included young female donors in our study. The donors can further be divided into three age subgroups (6–9 months, 12–14 months and 36–38 months) decently representing young canines.

The applied criteria hold both an advantage and a disadvantage, as age up to 3 years holds a significant timespan in canine species while at the same time, dogs within that age frame can be considered as young. Furthermore, we decided to characterise cAD-MSCs from young canines because it is important from the banking aspect as the elective surgical procedure is the opportunity to collect the sample and bank the cells for use in adult age.

#### 4.2.1. Adipose Tissue Collection

Adipose tissue was collected as medical waste after surgery. The origin of adipose tissue was the periovarian area, mesentery or falciform ligament (Table 1).

The dogs were sedated with an intramuscular cocktail of 0.3 mg/kg methadone (Comfortan, Genera dd, Croatia) and 2–3 µg/kg dexmedetomidine (Dexdomitor, Zoetis, Parsippany-Troy Hills, NJ, USA). Upon the onset of sedation, an intravenous catheter was placed in the antebrachial vein, and 2–5 mg/kg intravenous propofol (Propofol 1% (10 mg/1 mL) MCT Fresenius emulsion for injection or infusion, Fresenius Kabi, Bad Homburg, Germany) was applied for induction of general anaesthesia. An endotracheal tube was placed in the trachea, and anaesthesia was maintained via an inhalational mixture of 1–2% isoflurane (Forane, Abbott, Chicago, IL, USA) and oxygen. Intraoperative analgesia was provided by applying a bolus dose of fentanyl (1 mg/kg) followed by continuous intravenous administration of fentanyl 0.2 mg/kg/min (Fentanyl injections, Janssen Pharmaceutica N.V., Antwerp, Belgium). Postoperative analgesia was provided with meloxicam (Movalis, Boehringer Ingelheim, Zagreb, Croatia) in an initial bolus dose of 0.2 mg/kg intramuscular followed by a dose of 0.1 mg/kg orally, once a day, for at least 3 days. The surgical site was strichotomised and antiseptically prepared by cleaning with a 2% chlorhexidine soap solution for 5 min (Plivasept pjenušavi, Pliva d.o.o., Zagreb, Croatia). The final skin preparation was completed by applying a light coat of antiseptic surgical solution (70% alcohol) with a spray bottle and allowed to air dry. The surgical site was draped, and a midline celiotomy was performed. After ovariectomy or ovariohysterectomy, the periovarian, mesenteric or falciform adipose tissue (3–10 g) was collected into a sterile Falcon tube (without medium) and placed into a refrigerator until transport to the laboratory.

#### 4.2.2. cAD-MSCs Isolation and Expansion

All collected samples were stored at 4 °C and subjected to isolation protocol within 2 h after sample collection. Isolation of the cAD-MSCs was performed according to the protocol we previously published [30] using 5–8 g of abdominal adipose tissue for isolation. Adipose tissue samples were washed with sterile PBS (in house reagent) with the addition of 1% penicillin/streptomycin antibiotic (p/s; Sigma-Aldrich, St. Louis, MO, USA), minced and placed in 0.2% collagenase type I solution (ThermoFisher Scientific, Waltham, MA, USA) in 50 mL sterile closed tube for digestion during 50 min at 37 °C, 5% CO_2_ and 95% humidity, briefly stirring every 10 min. After the incubation period, foetal bovine serum (10%) (FBS, ThermoFisher Scientific, Waltham, MA, USA) was added to digested tissue to block collagenase activity; the suspension was filtered through 70 μm cell strainer (BD Biosciences, Franklin Lakes, NJ, USA) and centrifuged (Hettich Rotina 420, Tutlingen, Germany) 5 min at 2000 rpm (1400× *g*). The cell pellet was resuspended in 10 mL Dulbecco’s Modified Eagle’s Medium (DMEM) Low Glucose (ThermoFisher Scientific, Waltham, MA, USA) and centrifuged a second time at the same conditions. Finally, pellet was resuspended in prewarmed 79% DMEM Low Glucose + 20% FBS + 1% p/s (basal medium) and incubated at 37 °C, 5% CO_2_, 95% humidity. The medium was changed 24 h later, and all nonadherent cells were eliminated.

#### 4.2.3. cAD-MSCs Cultivation

Confluent, adherent cells were designated P0. Passaging was performed in a T25 cell culture flask (Nunc, ThermoFisher Scientific, Waltham, MA, USA) using the basal medium. All experiments were performed on freshly, not thawed cells cultivated in vitro and continuously passaged until proliferation arrest. Passaging was performed at a confluence of 80% to 90% up to proliferation arrest. A portion of cells was cryopreserved in P2 and P3 in basal medium + 10% DMSO (Sigma-Aldrich, St. Louis, MO, USA) at −80 °C using an alcohol-free freezing container (Corning, Corning, NY, USA) and then placed in liquid nitrogen. The cAD-MSCs suspensions were culture-negative for bacteria and fungi and polymerase chain reaction (PCR) was negative for *Mycoplasma* spp.

### 4.3. Differentiation Assay

Cells in P2 were passaged and used for the differentiation experiments as previously described [20] with significant improvements related to adipocytes and chondrocyte detection. Cells were induced to differentiate toward trilineage (adipogenic, osteogenic and chondrogenic). In brief, adipodifferentiation and osteodifferentiation tests were performed using a 24-microwell plate (Nunc, ThermoFisher Scientific, Waltham, MA, USA) by seeding 5 × 10^4^ cells per well in a basal medium. After 48 h, cells reached confluence and the basal medium was aspirated and 1 mL of StemMACS AdipoDiff (MIltenyi Biotec, Bergisch Gladbach, Germany). Media for adipocytes, StemMACS OsteDiff. Media for osteoblasts, (Miltenyi Biotec, Bergisch Gladbach, Germany) were added to particular wells except for control wells which were further cultivated in basal medium. Differentiation and basal media were changed every 48–72 h, and plates were microscopically (Zeiss, Oberkochen, Germany) examined (×10). Differentiation was performed for 21 days.

Chondrodifferentiation was performed in 15 mL conical polypropylene tubes with a cap according to Milteny Biotech protocol with minor modifications. In total, 10^5^ cells resuspended in the basal medium was added to the control and test tube and centrifuged at 235× *g* for 10 min. Basal medium was aspirated from the test tube for chondrodifferentiation, cells were resuspended in 1 mL of ChonroDiff Media (Milteny Biotech, Bergisch Gladbach, Germany) and the centrifugation step was repeated. Control tube containing cell pellet in basal medium and chondrodifferentiation test tube caps were slightly opened, and tubes were incubated at 37 °C, 5% CO_2_, 95% humidity 21 days. Media were changed every 48–72 h.

#### 4.3.1. Detection of Adipocytes

Detection of adipocytes was performed by removing StemMACS AdipoDiff Media and washing the cells twice with 300 μL of sterile PBS (in house reagent). Cells were fixed with 300 μL of 10% buffered formalin and incubated for 10 min at room temperature (RT). Formalin was aspirated completely, cells were washed twice with sdeionised H_2_O and 300 μL of Oil Red O (Sigma-Aldrich, St. Louis, MO, USA) was added to all wells. Plates were incubated for 20 min at RT. Oil Red O (Sigma-Aldrich, St. Louis, MO, USA) was aspirated, cells were washed 2× with sdeionised H_2_O and finally, 100 μL of deionised H_2_O was added to keep cells moisture. Immediately after staining, cells were examined under a microscope and pictures were taken with the camera (Zeiss, Oberkochen, Germany). Red colour stained cells were considered to be positive.

#### 4.3.2. Detection of Osteoblasts

Detection of osteoblasts was performed by removing StemMACS OsteoDiff. Media (Miltenyi Biotec, Bergisch Gladbach, Germany), and washing the cells twice with 300 μL of sterile PBS (in house reagent). Cells were fixed by adding 300 μL 10% buffered formalin and incubated 10 min at RT. Formalin was aspirated completely, cells were washed twice with deionised H_2_O and 300 μL of SIGMA*FAST* BCIP/NBT substrate (Sigma-Aldrich, St. Louis, MO, USA) was added to all wells. Plates were incubated for 10 min at RT. The substrate was aspirated, cells were washed 2× with deionised H_2_O and finally, 100 μL of deionised H_2_O was added to keep cells moisture. Immediately after staining, stained cells were examined under a microscope and pictures were taken with the camera (Zeiss, Oberkochen, Germany). Purple-stained cells were considered to be positive.

#### 4.3.3. Detection of Chondrocytes

Detection of chondrocytes was performed by carefully removing StemMACS ChondroDiff. Media (Miltenyi Biotec, Bergisch Gladbach, Germany) without aspirating spheroids and washing the spheroids twice with 300 μL of sterile PBS (in house reagent). Spheroids were carefully aspirated and placed in wells in a 24-microwell plate. Spheroids were fixed by adding 300 μL of neutral buffered formalin (3.7%) (in-house reagent) and incubated for 16 h at RT in the dark. Formalin was aspirated, and spheroids were washed twice with deionised H_2_O. Spheroids were dehydrated in ethanol series (2 × 30 min 70% ethanol, 2 × 30 min 80% ethanol, 2 × 30 min 96% ethanol, 2 × 30 min 100% ethanol) and placed in xylol.

Alcian Blue 8GX (Sigma-Aldrich, St. Louis, MO, USA) stain was used to detect proteoglycan aggrecan in the extracellular matrix (ECM) produced by chondrocytes in 3D micromass culture (i.e., chondrocyte nodule or spheroid) of cAD-MSCs cultivated for 21 days in StemMACS ChondroDiff Media (Miltenyi Biotec, Bergisch Gladbach, Germany). Only cAD-MSCs spheroids from the same donor cultivated in StemMACS Expansion Media were used as a negative control. Counterstaining was performed with Nuclear fast red-aluminium sulfate solution 0.1% (Sigma Aldrich, St. Louis, MO, USA), which stains cell nuclei pink to red.

Formalin-fixed paraffin-embedded spheroids were sectioned at 5 µm thickness and transferred onto positively charged glass slides.

After deparaffinisation in xylene (3 × 5 min.) and rehydration in decreasing ethanol concentrations (100% 5 min., 96% 5 min., 75% 5 min.) ending with deionised H_2_O (2 min.) slides were immersed in 3% acetic acid for 3 min, then in 1% Alcian Blue (Sigma-Aldrich, St. Louis, MO, USA) for 30 min, followed by rinsing a few times in 3% acetic acid and immersing in running tap water (10 min). Slides were rinsed a few times in deionised H_2_O before and after counterstaining with Nuclear fast red-aluminium sulfate (Sigma-Aldrich, St. Louis, MO, USA). After dehydration in increasing ethanol concentrations (70% and 96% 1 min., 100% 2 × 1 min.) and coverslipping, the slides were examined under a microscope Nikon Eclipse E600 (Nikon, Tokyo, Japan) and photographs were taken with Olympus DP20 (Olympus, Tokyo, Japan) camera.

### 4.4. Immunophenotyping

To reveal the expression pattern and percentage of positive cells for selected markers, cAD-MSCs were analysed in passages P1 to P6. For the flow cytometry experiments, we created an antibody panel (Table 3) based on the following criteria: antibodies have to be raised against a canine antigen or have to be cross-reactive with canines; antibodies have to be labelled with an appropriate fluorophore that can be detected with two BD FACSVerse (serial number Z6511540253, BD Biosciences, Franklin Lakes, NJ, USA) lasers (blue or red) and there has to be an appropriate isotype control with the same fluorophore as the belonging antibody.

Prior to each experiment, daily performance QC was performed using CS & T beads (BD Biosciences, Franklin Lakes, NJ, USA) lot number 92,323 and characterisation QC was performed every 6 months.

Cells were detached using a cell scraper and carefully resuspended and centrifuged at 235× *g* for 10 min. The medium was aspirated, and cells were resuspended in a 1 mL cell wash (BD Biosciences, Franklin Lakes, NJ, USA). Cell number was obtained using an automated cell counter (Corning, Corning, NY, USA). Cell number was adjusted to 10^5^ per mL, and 1 mL of cell suspension was carefully added to each test tube designated to antibody and isotype control from the panel. Tubes were centrifuged for 5 min at 235× *g*, the supernatant was aspirated and the cell pellet was carefully resuspended to enable antibodies to reach each cell. Antibody concentration was used according to manufaturers’ instructions. Single stained test tubes were incubated for 30 min at 4 °C. After the incubation period, 2 mL cell wash (BD Biosciences, Franklin Lakes, NJ, USA) was added and tubes were centrifuged for 5 min at 235× *g* to remove the excess of unbound antibodies. Finally, 500 µL of cell wash was added for flow cytometric analysis.

Experimental settings were set up using unstained cells, and the same settings were used for all tubes in each experiment. Cell viability was checked with Propidium Iodide staining solution (BD Biosciences, Franklin Lakes, NJ, USA) at the end of the experiment.

The same gating strategy has been used for all data files. The first gate (P1) was set for the selection of a homogenous population with the exclusion of cell debris using forward and side scatter parameters. The second gate (P2) was set using forward-scatter height and area parameters for double cell exclusion, so only singlets were selected for further analysis.

Results were analysed using FACSuite software. The results for 10,000 acquired events were expressed as median fluorescence intensity (MFI). MFI fold change was calculated by dividing the median of the appropriate antibody with the median of isotype control ($\frac{MFI\;Antibody}{MFI\;Isotype\;control}$). Obtained values (from each donor for each marker) were used to calculate the average, which was further used to present data graphically. The average MFI and standard deviation for each CD of all donors in each passage were calculated and data were graphically presented.

### 4.5. Gene Expression

Changes in gene expression of cAD-MSCs markers (CD90^+^, CD44^+^, CD73^+^, CD29^+^, CD105^+^ and CD45^−^) were investigated using validated RT^2^ Profiler PCR Array Format R (Qiagen, Hilden, Germany) suitable for use with Rotor-Gene Q (Qiagen, Hilden, Germany). This array includes SYBR Green-optimized primer assays.

#### 4.5.1. Total RNA Extraction

Total RNA was isolated from cAD-MSCs cultivated in a T75 cell culture flask. Cells were detached using a cell scraper, centrifuged at 235× *g* for 10 min and used immediately for RNA isolation. The RNA was isolated using the RNeasy Mini Kit (Qiagen, Hilden, Germany), following the manufacturer’s instructions. The integrity of isolated RNA was examined by 1% agarose gel electrophoresis; the RNA concentration and purity were determined by measuring the absorbance in a Nanophotometer P360 (Implen, Munich, Germany).

#### 4.5.2. Real-Time PCR Array

RT^2^ First Strand Kit (Qiagen, Hilden, Germany) was used for genomic DNA (gDNA) elimination and cDNA synthesis, which served as a template for RT^2^ Profiler PCR Array (Qiagen, Hilden, Germany). cDNA was synthesised from 800 ng of total RNA. First, in a 10 μL reaction, gDNA was eliminated for 5 min at 42 °C and then the mixture was used in a 20 μL cDNA synthesis mixture according to the manufacturer’s instructions.

Cycling conditions were set according to the manufacturer’s instructions. The array contains primers specific for CD90, CD44, CD73, CD29, CD45 and CD105, and primers for gDNA, RT and PCR controls. One well is used to check gDNA control, 3 wells contain reverse-transcription controls and 3 wells contain a positive PCR control. Obtained data were analysed using RT^2^ Profiler PCR Array Data Analysis software available online: https://dataanalysis2.qiagen.com/pcr (accessed on 3 February 2021). The software analyses the data using a ΔΔCt method and performs statistical analysis of the data (based on Student’s *t*-test); differences between gene expression levels were considered significant when *p* < 0.05. Fold change cutoff of 2 was chosen.

### 4.6. Statistics

The data distribution was checked with the Kolmogorov–Smirnov test. To investigate statistical significance between mean values obtained in early passages (P1–P3) and late passages (P4–P6), we used Student’s *t*-test. For graphical representation, we used the Whisker box plot to reveal minimum, maximum, median and quartiles for MFI fold change values of each marker from P1 to P6.

## Figures and Tables

**Table 1:** Changes in gene expression between passage 3 and passage 6.

Symbol	AVG Ct		Standard Deviation		AVG Delta(Ct) (Ct(GOI)—Ave Ct(HKG))		Standard Deviation		2^(−Avg.(Delta(Ct)))^		Fold Change (Comparing to Control Group)	*p*-Value (Comparing to Control Group)	Up–Down Regulation (Comparing to Control Group)
	P3	P6	P3	P6	P3	P6	P3	P6	P3	P6	P6	P6	P6
CD44	19.36	16.99	6.716420	1.548428	4.56	0.76	7.026048	2.686248	0.042306	0.588725	13.92	0.337119	13.92
CD105	20.39	22.01	3.632936	5.497423	5.59	5.78	3.893338	7.179577	0.020694	0.018228	0.88	0.345515	−1.14
CD29	16.54	15.13	3.526914	1.812936	1.74	−1.10	3.361122	2.769505	0.299093	2.147017	7.18	0.331704	7.18
CD73	23.74	25.84	4.609488	4.044292	8.94	9.61	4.350983	3.909274	0.002037	0.001282	0.63	0.507992	−1.59
CD45	27.65	27.69	1.551907	6.902877	12.85	11.46	4.233130	6.742563	0.000135	0.000356	2.62	0.368872	2.62
CD90	17.11	21.58	3.441485	4.568415	2.31	5.35	1.028564	8.537819	0.201242	0.024473	0.12	0.348287	−8.22

**Table 2:** Donor data.

Donor	Species	Age (Months)	Breed	Surgical Procedure	Anatomical Origin of Adipose Tissue Used as a Source of cAD-MSCs	Health Status before Procedure
4/20	Canine	12	Medium poodle	Ovariohysterectomy	Mesenteric	No detectable signs of illness
5/20	Canine	14.5	Ridgeback	Ovariotomy	Periovarian area	No detectable signs of illness
6/20	Canine	6	Dachshund short-haired	Ovariotomy	Periovarian area	No detectable signs of illness
7/20	Canine	9	Mixed breed	Ovariotomy	Periovarian area	No detectable signs of illness
8/20	Canine	9	Beagle	Ovariotomy	Ligament falciform	No detectable signs of illness
9/20	Canine	9	Jack Russell Terrier	Ovariotomy	Periovarian area	No detectable signs of illness
11/20	Canine	36	Belgian Shepherd	Gastropexy	Ligament falciform	No detectable signs of illness
13/20	Canine	38	Mixed breed	Ovariotomy	Ligament falciform	No detectable signs of illness

**Table 3:** Antibody panel created for the study.

Antigen	Clone	Host	Fluorophore	Reactivity	Clonality	Manufacturer/Serial Number
CD44	MEM-263	Mouse	FITC	Canine, Human, Porcine	Monoclonal	Antibodies-online, Germany/ABIN452099
Isotype IgG1	VI-AP	Mouse	FITC		Monoclonal	Antibodies-online, Germany/ABIN1741583
CD45	YKIX716.13	Rat	PE	Canine	Monoclonal	Bio-Rad, USA/MCA1042PE
Isotype IgG2b		Rat	PE		Monoclonal	Bio-Rad, USA/MCA6006PE
CD73		Rabbit	PE	Human, Mouse, Rat, Dog, Chicken	Polyclonal	Bioss antibodies, USA/bs-4834R-PE
Isotype IgG		Rabbit	PE		Polyclonal	Antibodies-online, Germany/ABIN376422
CD 29	MEM-101A	Mouse	FITC	Canine, Human, Porcine	Monoclonal	Antibodies-online, Germany/ABIN94056
Isotype IgG1	VI-AP	Mouse	FITC		Monoclonal	Antibodies-online, Germany/ABIN1741583
CD271	ME20.4-1.H4	Mouse	FITC	Human, Canine	Monoclonal	Miltenyi Biotec, Germany/130-098-103
Isotype			FITC		Monoclonal	Miltenyi Biotec, Germany/130-113-761
CD90	5E10	Mouse	APC	Human, cross-reacts with monkey, porcine, canine protein	Monoclonal	Covalab, France/mab21094
Isotype IgG1	Unknown	Mouse	APC		Monoclonal	Antibodies-online, Germany/ABIN2145334
CD105	SN6	Mouse	PE-Cy 7	Human, published species canine	Monoclonal	Thermo Fisher Scientific25-1057-42
CD14		Mouse	PE-Cy 7	Human (QC Testing), Dog (Tested in Development)	Monoclonal	BD Pharmingen
Isotype IgG2		Mouse	PE-Cy 7		Monoclonal	BD Pharmingen

## Data Availability

The datasets used and/or analysed within the frame of the study can be provided by the corresponding author upon reasonable request.
